# Simulating Federated Learning to Enable Multi‐Hospital Collaboration for Lumbopelvic Alignment Estimation

**DOI:** 10.1002/jsp2.70120

**Published:** 2025-10-16

**Authors:** Andrea Cina, Miklovana Tuci, Ferran Pellisé, Caglar Yilgor, Ahmet Alanay, Javier Pizones, Frank Kleinstück, Ibrahim Obeid, Yann Philippe Charles, Sarah Richner‐Wunderlin, Fabio Galbusera, Catherine R. Jutzeler, European Spine Study Group

**Affiliations:** ^1^ Department of Health Sciences and Technology (D‐HEST), ETH Zurich Universitätstrasse 2 Zürich Switzerland; ^2^ Schulthess Clinic, Department of Teaching, Research and Development Zürich Switzerland; ^3^ Swiss Institute of Bioinformatics (SIB) Lausanne Switzerland; ^4^ Spinal Cord Injury Center University Hospital Balgrist, University of Zürich Zürich Switzerland; ^5^ Spine Research Unit Vall d'Hebron Research Institute Barcelona Spain; ^6^ Spine Surgery Unit Vall d'Hebron University Hospital Barcelona Spain; ^7^ Department of Orthopedics and Traumatology Acibadem University Istanbul Turkey; ^8^ Spine Surgery Unit La Paz University Hospital Madrid Spain; ^9^ Spine Center Division Schulthess Clinic Zurich Switzerland; ^10^ Spine Surgery Unit Bordeaux University Hospital Bordeaux France; ^11^ Department of Spine Surgery University Hospital of Strasbourg Strasbourg France

**Keywords:** automatic assessment, deep learning, federated learning, privacy, spine parameters

## Abstract

**Background:**

Accurate computation of radiological parameters related to spinal alignment is clinically crucial for diagnosing and managing conditions, such as adolescent idiopathic scoliosis and adult spinal deformities. Key parameters, including sacral slope, pelvic tilt, pelvic incidence, and lumbar lordosis, are required to assess lumbosacral alignment. Artificial Intelligence (AI) has demonstrated strong potential in automating these assessments, reducing clinician workload and improving consistency. However, AI models require large, diverse, high‐quality datasets to perform reliably across different clinical settings. Privacy concerns and data ownership issues often hinder data sharing, limiting the creation of centralized datasets.

**Methods:**

In this study, we demonstrate that federated learning (FL) enables the training of deep learning models across four hospitals without compromising patient privacy. In particular, we compared FL against a centralized approach, where data from all the hospitals are pooled together and a model is trained on them, and a local approach consisting of training individual models exclusively on data from each respective hospital, resulting in distinct hospital‐specific models.

**Results:**

FL achieved performance comparable to centralized training (errors ~5°), where data is pooled, and consistently outperformed models trained on data from individual hospitals, both in internal (~8°) and external (~10°) testing.

**Conclusion:**

This work highlights FL as a viable solution for collaborative AI development in spinal imaging, facilitating the use of diverse, multi‐institutional data while circumventing privacy barriers and complex data‐sharing agreements. Additionally, FL demonstrates particular benefits for smaller hospitals, enabling them to achieve superior model performance by effectively leveraging data from hospitals with larger datasets.

## Graphical abstract

Federated learning (FL) effectively estimates lumbopelvic parameters from multi‐center spinal imaging, matching centralized methods' accuracy while preserving patient privacy, highlighting FL's potential to enhance diagnostic precision and broaden AI‐driven clinical applications in spine care without compromising data confidentiality.

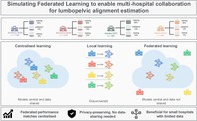

## 1 Introduction

The rise of Artificial Intelligence (AI) and the digitization of healthcare data have created unprecedented opportunities to develop tools that enhance diagnostic accuracy and clinical efficiency [1, 2, 3]. The spine field is not an exception. Integrating AI into radiological image analysis for spinal disorders has significantly advanced diagnostic precision, treatment planning, and prognostic evaluation. Among various AI applications, Convolutional Neural Networks (CNNs) have been widely employed to automatically assess the severity of scoliosis and the sagittal alignment of the spine from radiographs [4, 5]. Numerous studies have reported high performance of deep learning (DL) models in automatically evaluating spinal parameters, demonstrating their effectiveness in retrospective analyses [6, 7, 8, 9]. These tasks, often requiring extensive manual annotation, would be labor‐intensive and time‐consuming without automation.

One of the fundamental requirements for developing a good‐performing DL model is the availability of large and diverse datasets. Even medical datasets containing thousands of images are considered small if compared to publicly available datasets, such as ImageNet (~14 million images) [10], Common Objects in Context (COCO) [11], or Open Images [12], which have driven advances in DL model developments [13, 14, 15, 16, 17]. Limited dataset sizes in medical imaging often result in poor generalizability, with models trained at a single institution frequently underperforming when applied to external data from other hospitals [18]. Therefore, rigorous external validation is essential to demonstrate the robustness of machine learning (ML) models prior to clinical implementation [19, 20]. A promising solution to the challenge of limited generalizability is the creation of large, shared datasets composed of medical images and annotations from multiple institutions. Such datasets would enable the training of DL models on diverse and representative samples, significantly enhancing their robustness and performance across clinical settings. However, the development of multi‐institutional datasets is severely constrained by data privacy regulations and concerns over data ownership. While data‐sharing agreements offer a pathway for collaboration, they are often difficult to negotiate, requiring substantial time, legal resources, and administrative effort. As a result, the lack of scalable and efficient data‐sharing frameworks remains a critical barrier to progress in medical AI.

**FIGURE 1:**
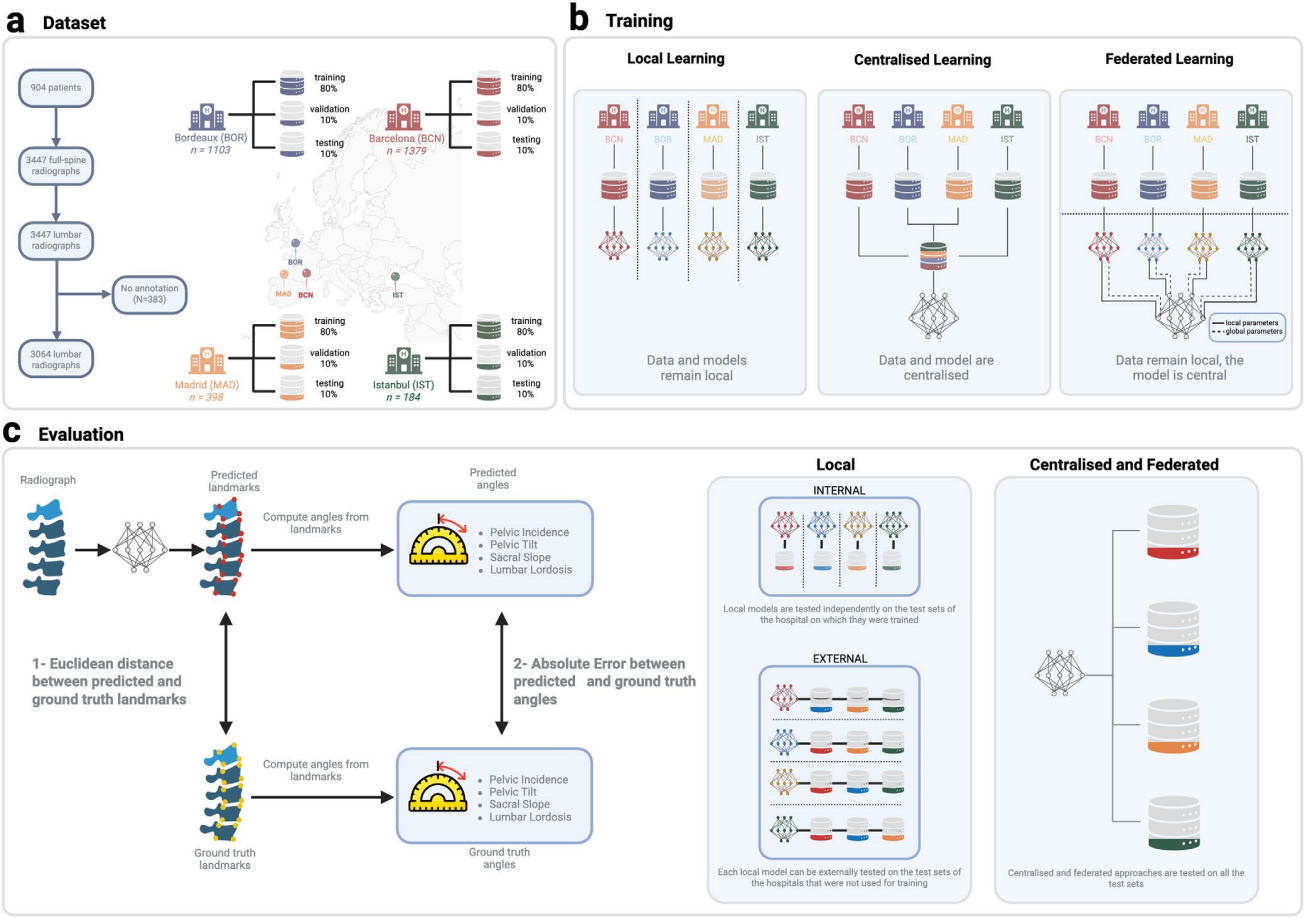
General workflow. (a) Each hospital is independently partitioned into 80% training, 10% validation and 10% held‐out test sets. (b) Three distinct training strategies are implemented: In the local setting, each site trains and tunes a model only on its own data; in the centralized approach, all sites pool training and validation sets in a unique dataset to develop one global model; in the federated approaches, each site alternates between (i) fitting its local model on private training data and sending only model parameter updates to a central model (solid lines), where they are aggregated and redistributed back (dashed lines) to every site using different strategies (FedAvg, FedProx, and FedOpt). (c) Schematic of the evaluation strategies: 1—vertebrae localization and 2—angles calculation. Each local model is first tested on its own institution's held‐out test set (internal validation) and then on the test sets of the three other hospitals (external validation). The centralized and federated approaches are evaluated similarly on every test set.

Over the last decade, federated learning (FL) emerged as a promising solution to the challenges of data privacy and sharing in medical AI. FL enables the training of DL models across multiple hospitals without requiring direct data exchange, thereby preserving patient privacy [21]. A growing body of work is exploring FL across multiple domains. For example, FL has been successfully applied in fields such as oncology (for glioblastoma boundary detection [22] and pediatric brain tumor analysis [23]), dermatology, and radiology [24, 25]. A recent review on FL in spine surgery [26] highlighted its potential to address challenges related to data heterogeneity and privacy. Applications include telesurgery, AI‐based outcome prediction, and vertebral segmentation. Despite these advances, comprehensive multi‐center validation studies evaluating the performance and clinical applicability of FL in spine imaging remain limited. Moreover, there is a lack of previous studies that have systematically compared centralized and single‐hospital training to various FL strategies (e.g., FedAvg [21], FedOpt [27], FedProx [28]) for spine imaging applications. To bridge this gap in the literature, we aimed to evaluate how FL performs in clinically meaningful scenarios by leveraging real‐world multi‐center data and focusing on parameters relevant to spinal alignment and surgical outcomes. Using data from four European hospitals, we conducted a simulation study using real‐world datasets to systematically evaluate the effectiveness of FL under controlled conditions, comparing FL against centralized training—where data from all hospitals are pooled into a single dataset—and local training, where models are trained independently at each hospital (Figure 1). Additionally, we investigated and compared three FL strategies—Federated Averaging (FedAvg) [21], Federated Optimization (FedOpt) [27], and FedProx [28]—to identify the approach providing optimal performance in terms of computational cost and ease of hyperparameters configuration.

## 2 Methods

### 2.1 Study Design and Dataset

This study evaluates the effectiveness of FL in computing key lumbopelvic parameters, including sacral slope (SS), pelvic incidence (PI), pelvic tilt (PT), and lumbar lordosis (LL), which are clinically essential for assessing spinal alignment and informing surgical decisions [29, 30]. To this end, we used a DL localization model designed to automatically identify anatomical landmarks on lateral lumbar x‐rays, from which these parameters can be derived. A large multi‐center dataset was collected to investigate the applicability of the FL approach to train this localization model in a privacy‐preserving manner. The dataset consisted of sagittal x‐ray images from four different institutions in Europe participating in the ESSG consortium (https://www.spine‐essg.com). The hospitals are located in Barcelona (Vall d'Hebron University Hospital), Madrid (La Paz University Hospital), Bordeaux (Pellegrin Bordeaux University Hospital), and Istanbul (Acibadem University Maslak Hospital). Each of the participating sites obtained institutional review board approval for patient enrollment and data collection protocols, and all patients provided informed consent. The study was conducted following the TRIPOD+AI guideline [31]. The images were full‐trunk radiographs annotated with anatomical landmarks ranging from vertebra C7 down to the femoral heads. To simulate lumbar radiographs, images were cropped by retaining the field of view from the femoral heads up to vertebra T12 and performing random cropping around the T10/T11 region. After cropping, each image included annotations for 29 anatomical landmarks from T12 to the upper endplate of the sacrum, along with the centers of the femoral heads. The sole inclusion criterion was the availability of a complete set of landmarks from T12 to the femoral heads: images without complete landmark visibility were excluded because they could not be reliably annotated. In particular, images were excluded when one or more landmarks were (i) outside the field of view (e.g., truncated femoral heads), (ii) obscured (e.g., overlap with instrumentations or other devices that prevented a reliable localization), or (iii) not reliably identifiable due to image quality (e.g., severe motion blur). No other exclusion criteria were applied to maximize clinical diversity (e.g., images with instrumentation and with slight motion artifact were retained). Therefore, the images encompassed various clinical scenarios, including instrumentation, mild to severe scoliosis, and degenerative conditions, making it challenging for the model to achieve high performance in parameter calculation. For the dataset split, we first grouped images by patient and center and then performed a center‐stratified random split at the patient level into training (80%), validation (10%), and test (10%) sets. All images from the same patient were assigned to the same split to prevent leakage. This procedure preserves per‐center proportions across splits.

The aim is to simulate a federated setting and compare it against (1) a centralized training and (2) a local training. The centralized training strategy involved pooling data from all participating hospitals into a single, centralized dataset, from which a global model was trained. In contrast, the local training strategy consisted of training individual models exclusively on data from each respective hospital, resulting in distinct hospital‐specific models. To explore collaborative alternatives, we implemented different FL approaches, applying the FedAvg [21] algorithm alongside two additional FL variants, FedOpt [27] and FedProx [28]. These methods enabled the integration of knowledge from each hospital's local dataset without directly sharing data. Indeed, the federated approaches aggregate model parameters shared by each hospital to form a unified global model while preserving data privacy. For all approaches, the best‐performing model parameters were selected based on validation set performance and subsequently evaluated on a held‐out test set. Notably, each locally trained model was assessed on both internal (same hospital) and external (other hospitals) test datasets to measure internal performance and cross‐site generalizability (Figure 1). This pipeline enabled a comprehensive comparison of centralized, local, and federated training strategies, facilitating an evaluation of their suitability for clinical deployment and potential performance benefits across diverse hospital environments.

### 2.2 Federated Learning

FL [21] is an emerging area of research that enables ML models to train a shared prediction model by distributing the training across different devices or hospitals while keeping the data private. In this study setting, the FL framework requires each hospital to act as a client and a central server to act as the clients' coordinator (Figure 1). The client's role is to train the model on its own data and periodically send the model's parameters to the central server. The server aggregates the model updates from all the clients and sends them back to each client. FedAvg [21], which is the simplest form of FL consisting of a weighted average (weighted based on the hospital's amount of images), was first used in our simulation (Figure 1). FedAvg was chosen as the primary baseline since it is the standard, widely used FL method offering a clear reference point with minimal assumptions and tuning.

In addition to the FedAvg approach, two alternative strategies were used: FedOpt [27] and FedProx [28]. FedOpt extends FedAvg by incorporating advanced optimization techniques at the server level, such as adaptive learning rates and momentum decays, to enhance the convergence of the global model. Meanwhile, FedProx introduces an additional proximal term into the local objective function that regularizes each client's training process. This proximal term helps to mitigate the impact of data heterogeneity by constraining local updates to remain closer to the global model, thereby promoting more stable convergence. These two FL approaches were selected to address two main challenges of our dataset: (1) presence of only two relatively large hospitals in each round, and (2) differences across centers in demographics, imaging protocols, and pathology. FedOpt was expected to reach a good validation performance in fewer rounds and to be less sensitive to learning rate than FedAvg, while FedProx was expected to reduce client drift and stabilize training for centers that differ the most from the overall distribution.

However, while FedAvg requires only minimal tuning (mainly specifying the number of local epochs and FL rounds), both FedOpt and FedProx involve additional hyperparameters such as learning rates, momentum factors, and the proximal coefficient in FedProx, making them more complex to tune. By incorporating all three approaches in our simulation, we aimed to assess not only their predictive performance but also their ease of configuration in a multi‐institutional, privacy‐preserving setting. Other FL approaches were not included to keep the manuscript more concise. Moreover, the selected methods were considered sufficient to cover some key design aspects, namely simple averaging and optimization‐based schemes.

### 2.3 Model Architecture and Training

**FIGURE 2:**
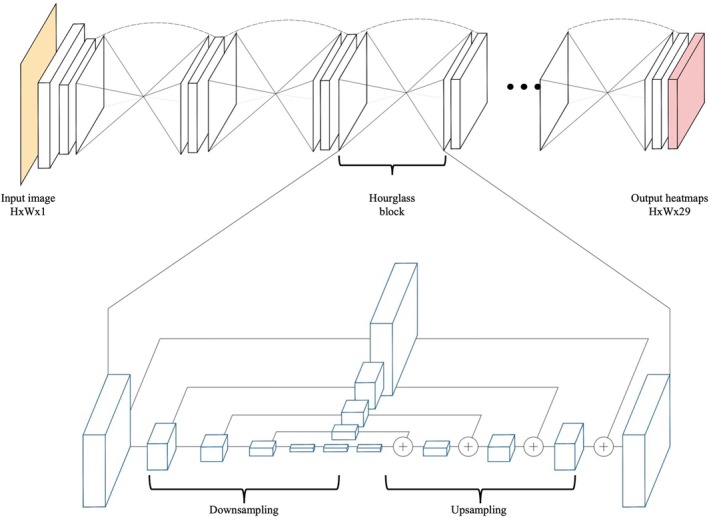
Hourglass architecture. At the top the input image (yellow) is processed by a stack of hourglass modules (two in our setting), producing 29 output heatmaps (red; one heatmap for each target keypoint). At the bottom, the structure of a single hourglass module, showing the symmetric downsampling/upsampling scheme with skip connections. Feature maps from corresponding resolutions are fused by element‐wise addition (+) before generating the final output.

To localize the 29 landmark coordinates, an Hourglass Network (Figure 2) [32] with two stacked hourglass modules was used since it has shown state‐of‐the‐art performance on many human pose estimation studies [33, 34, 35]. On top of the last convolutional layer feature map, the DSNT layer [36] that was already implemented in [5, 37] was used to perform the coordinates regression using a spatial‐to‐numerical transformation from the spatial heatmaps. The images were scaled to 768 × 768 resolution using bicubic interpolation and preserving the aspect ratio. Several augmentation techniques such as CLAHE, coarse dropout, brightness and contrast correction, blurring, noise, and random rotations (−10° to +10°) were applied to increase the robustness of the model during training. The augmentation was fixed for all the simulations to allow a fair comparison. The model was trained using the Euclidean distance between the predicted and the ground truth points as the loss, plus a regularization term that computes the divergence between the heatmaps generated from the ground truth coordinates and the heatmaps predicted by the model [36] (Equation 1). The heatmaps can be seen as probability distributions of the points' locations. The loss is calculated as

(1) $$L={\left\|p-\mathrm{gt}\right\|}^{2}+D\left({\mathrm{hm}}_{\mathrm{gt}},{\mathrm{hm}}_{p}\right)$$

where *p* represents the predicted coordinates, *gt* the ground truth coordinates, ${\left\|.\right\|}^{2}$ is the Euclidean distance, and *D* is the divergence between the ground truth heatmaps (${\mathrm{hm}}_{\mathrm{gt}}$) and the predicted heatmaps (${\mathrm{hm}}_{p}$).

**FIGURE 3:**
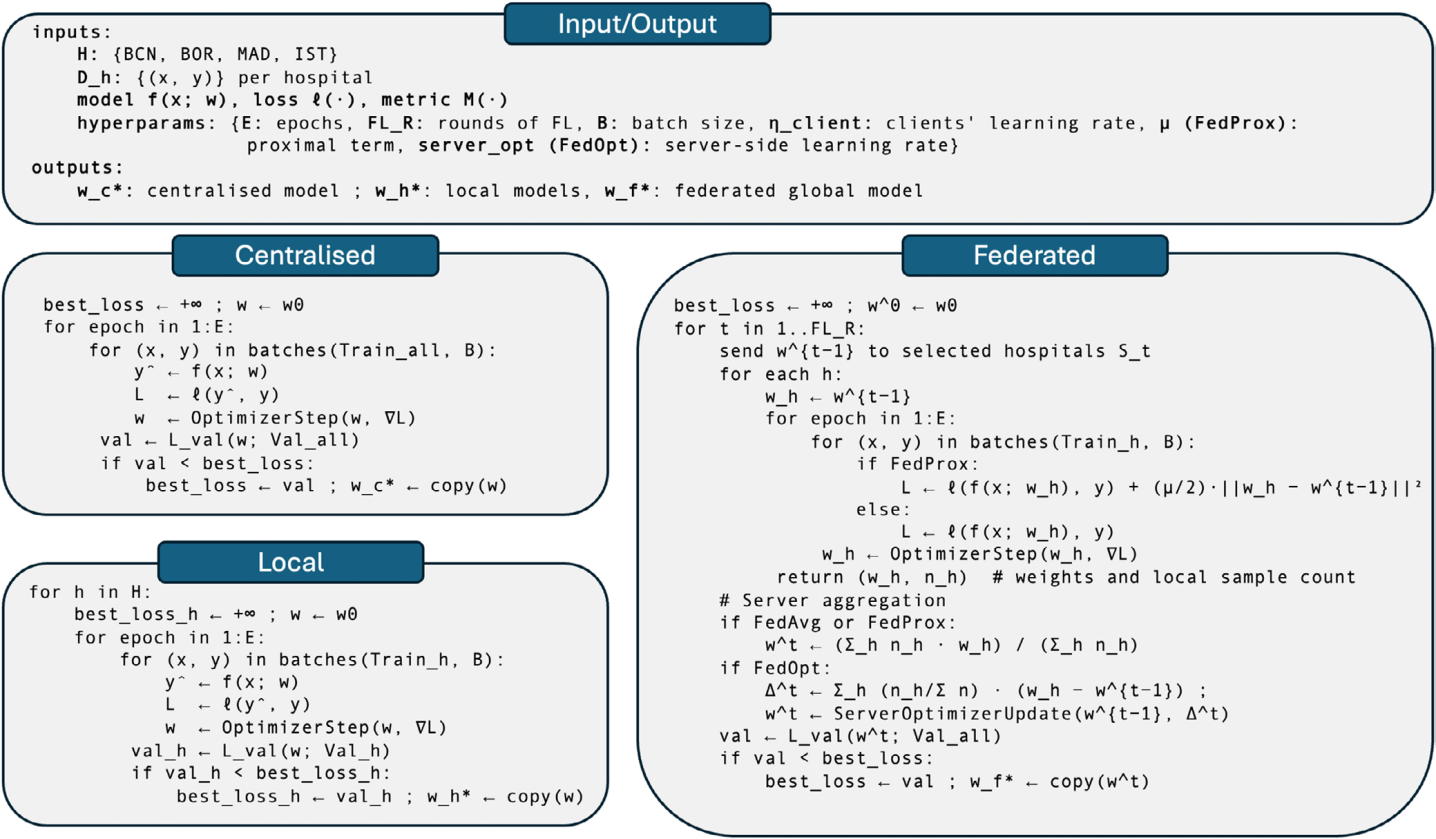
Algorithmic overview of the training pipelines. The panel summarized input/output and the three strategies: Centralized (single model trained on pooled data with validation checkpointing), local (one model per hospital trained on its own data), and federated (FL rounds and different aggregation strategies depending on the method).

Regarding the training procedures (Figure 3), in the centralized approach, the full training dataset was used to train a single global model for 200 epochs, with the validation set serving as a checkpoint to select the best‐performing model; similarly, in the local approach, each hospital trained its own model on only its proprietary subset of the training data for 200 epochs, also using the validation set to determine the optimal model checkpoint. The learning rate was set to 0.0001 for both approaches and was reduced by a factor of 10 after 50 epochs. Early stopping was applied if no improvement was observed on the validation set for 10 consecutive epochs. In the FedAvg setup, each hospital trained its local model for 10 epochs before sending the model parameters to a central server, which computed an aggregated update via the FedAvg method; the updated model was then redistributed to the hospitals, where local training resumed for another 10 epochs (Figure 1). This process was repeated for 30 rounds. The learning rate was set to 0.0001 and reduced by a factor of 10 after 15 rounds of FL. Following the suggestions in [27] and after experimenting with some numbers, FedOpt was configured to run 3 local epochs over 100 global rounds, whereas FedProx was set to 10 local epochs over the same number of global rounds. For FedOpt, the local and global learning rates were 0.001 and 0.0001, respectively. For FedProx, the local learning rate was 0.0001 and the global learning rate was 0.00001. The batch size was set to 8 for all the simulations (see Table 1 for details).

**TABLE 1:** Training hyperparameters for the centralized, local, and federated pipelines.

Strategy	Local epochs per round	Global rounds	Total local epochs per client	Learning rate(s)	LR schedule	Batch size
Centralized	—	—	200	0.0001	×0.1 after 50 epochs	8
Local	—	—	200	0.0001	×0.1 after 50 epochs	8
FedAvg	10	30	300	0.0001 (client)	×0.1 after 15 FL rounds	8
FedOpt	3	100	300	0.001 (client), 0.0001 (server)	—	8
FedProx	10	100	1000	0.0001 (client), 0.00001 (server)	—	8

### 2.4 Evaluation

The Euclidean distance between predicted and ground truth landmark locations was computed to assess the model's localization performance. In particular, point plots with 95% confidence intervals were used to analyze the performance of the different approaches on each vertebra point localization from T12 to the femur heads. Then, the lumbosacral parameters were computed from the ground truth and predicted landmarks on the test set. In particular, SS, PI, PT, and LL were calculated. The performance was evaluated as the absolute difference between the ground truth and predicted parameters. This evaluation was conducted on the test set for each parameter by first pooling data across all hospitals and then analyzing the performance separately for each hospital, potentially identifying any variations in model performance across institutions. The evaluation was assessed for the centralized, federated, and local approaches both internally—using a hospital's own test data—and externally—using data from other hospitals—to highlight any inter‐hospital variations in predictive performance. Boxplots were used to represent error distributions. To further evaluate the accuracy of the predicted angles, we computed the percentage of correct predictions (PCPs), meaning the percentage of predictions falling within various error thresholds (from 1° to 15°). Specifically, for each angle, the proportion of samples whose predicted values differed from the ground truth by less than or equal to a given threshold was calculated. This metric provides insight into the distribution of prediction accuracy at incremental tolerance levels.

All the analyses and plots were performed using Python 3.11.6 with pandas, numpy, matplotlib, and seaborn libraries. The DL model was implemented in PyTorch using the DSNT library for the DSNT layer. The FL framework was implemented using the Flower library. The model was trained on the ETH HPC LeoMed on a 24 GB GPU RTX 3090.

## 3 Results

### 3.1 Dataset

Of the initial dataset, 383 images were excluded because at least one required landmark was not visible or could not be placed with sufficient confidence (e.g., truncated femoral heads, severe motion blur). The final dataset comprised 3064 lateral spine radiographs from 904 patients across four institutions, each with 3–4 follow‐up visits. Images were cropped from full‐trunk x‐rays to focus on the lumbar region. The cohort had a mean age of 52.5 years (SD = 18), a mean BMI of 24.5 (SD = 4.6), and was predominantly female (80%). Idiopathic (56%) and degenerative (30%) scoliosis were the most common diagnoses, with additional cases including congenital, neuromuscular, and Scheuermann's disease. Patient demographics varied slightly by center: IST had a younger cohort (mean age 35), while BOR, BCN, and MAD ranged from 51 to 57 years. Pathology profiles were similar across BOR, BCN, and MAD, with idiopathic (41%–69%) and degenerative (25%–50%) cases dominating. In contrast, IST featured a more diverse pathology mix, including 15% congenital and 11% Scheuermann's disease, with only 9% degenerative cases. The dataset was split into training (*N* = 2435), validation (*N* = 318), and test (*N* = 311) sets, ensuring no patient overlap across splits. Hospital contributions were 45% (BCN), 35% (BOR), 14% (MAD), and 6% (IST) for each set (see Table 2 for details).

**TABLE 2:** Dataset split and image counts per center.

Split	Total *N*	BCN	BOR	MAD	IST
Train	2435	1096	861	324	154
Validation	318	147	113	44	14
Test	311	141	109	43	18
Total	3064	1379	1104	397	184

### 3.2 Evaluation

The performance of each approach was assessed across multiple tasks. First, vertebral localization accuracy was evaluated to provide an initial indication of the model's ability to correctly identify vertebral positions. Subsequently, spinal parameter calculations were evaluated at various levels of granularity, including a general analysis of parameter estimation errors, a hospital‐specific performance analysis, an evaluation across different tolerance thresholds, and a detailed comparative analysis of the FL‐based approaches. Regarding computational time, the centralized model required 22 h to train. Local models trained in 10 h (BCN), 7.7 h (BOR), 2.9 h (MAD), and 1.4 h (IST). In the FL settings, a single epoch took approximately 180 s (BCN), 120 s (BOR), 45 s (MAD), and 25 s (IST). FedOpt had a small additional overhead due to server‐side optimization, but this was negligible compared to client‐side time.

#### 3.2.1 Vertebral Body Localization

**FIGURE 4:**
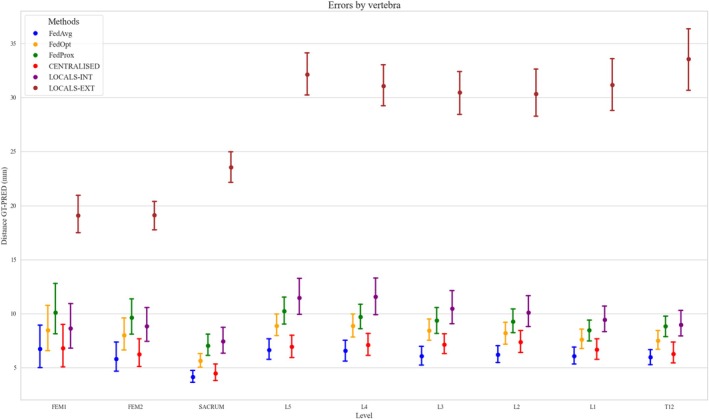
Errors by vertebra. Error bars for each vertebra (from the sacrum up to T12) under the four training approaches: Centralized, Federated, Locals‐int, and Locals‐ext. The vertical axis represents the distance or deviation in millimeters from the ground‐truth vertebra positioning. This visualization highlights the method‐specific performance across different levels of the spinal column.

In terms of Euclidean distance between predicted and ground‐truth landmark locations, both the centralized and FedAvg models achieved similarly low localization errors—typically under 9 mm for all levels. The sacrum was the best predicted, with median localization errors below 4 mm and small error variability. In contrast, local models showed markedly higher errors, especially from T12 to the sacrum, and performed poorly in external validation (Figure 4). The Euclidean distances between ground truth and predicted positions from T12 to L4 were above 30 mm in the external validation of local models. Even in internal evaluations, local models underperformed relative to both centralized and FedAvg models, with median errors exceeding 5 mm considering levels from T12 to L5. For the sacrum and the femoral heads, the errors were below 10 mm. FedOpt and FedProx also trailed behind centralized and FedAvg approaches, with median errors around 10 mm, though they consistently outperformed local models, particularly in the T12–L5 region.

#### 3.2.2 Comparative Performance of Learning Strategies in Spinal Parameter Prediction

**FIGURE 5:**
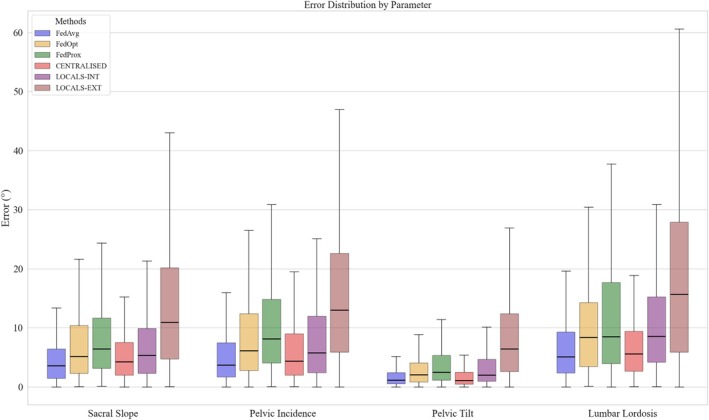
Error distribution by parameter. Comparison of the estimation errors (in degrees) for four spinal parameters (sacral slope, pelvic incidence, pelvic tilt, and lumbar lordosis) across the six training approaches: FedAvg, FedOpt, FedProx, Centralized, Locals‐int (local models tested on the internal test set), and Locals‐ext (local models tested on other hospitals' data).

FedAvg matched the performance of the centralized model while consistently outperforming local models—particularly in cross‐site (external) evaluations (Figure 5). In contrast, the more complex FL variants, FedOpt and FedProx, failed to surpass the simpler FedAvg. Across all key lumbopelvic parameters (SS, PI, PT, LL), both the centralized and FedAvg models achieved low mean absolute errors (MAEs), typically between 4° and 6°, underscoring their clinical viability. For example, FedAvg achieved an MAE of 5.2° (SD = 6.4°) for sacral slope (SS), outperforming both the centralized model (6.6°, SD = 11.5°) and local models, which reached 15.9° (SD = 18.4°) when tested externally. Similar patterns were seen across other parameters: for pelvic incidence (PI), FedAvg recorded 5.8° (SD = 6.5°), compared to 6.7° (SD = 7.4°) for the centralized model and up to 16.0° (SD = 12.8°) for externally tested local models. Pelvic tilt (PT) showed the lowest errors overall, with FedAvg and the centralized model achieving 2.2° (SD = 3.4°) and 2.5° (SD = 5.2°), respectively, while local models lagged behind. For LL, FedAvg reached 7.1° (SD = 6.8°), again outperforming local models, which showed errors exceeding 19° in external evaluations. Taken together, these findings highlight FedAvg as a robust and scalable alternative to centralized learning, offering high accuracy without compromising patient data privacy—key for real‐world clinical deployment.

#### 3.2.3 Hospital‐Specific Analysis

**FIGURE 6:**
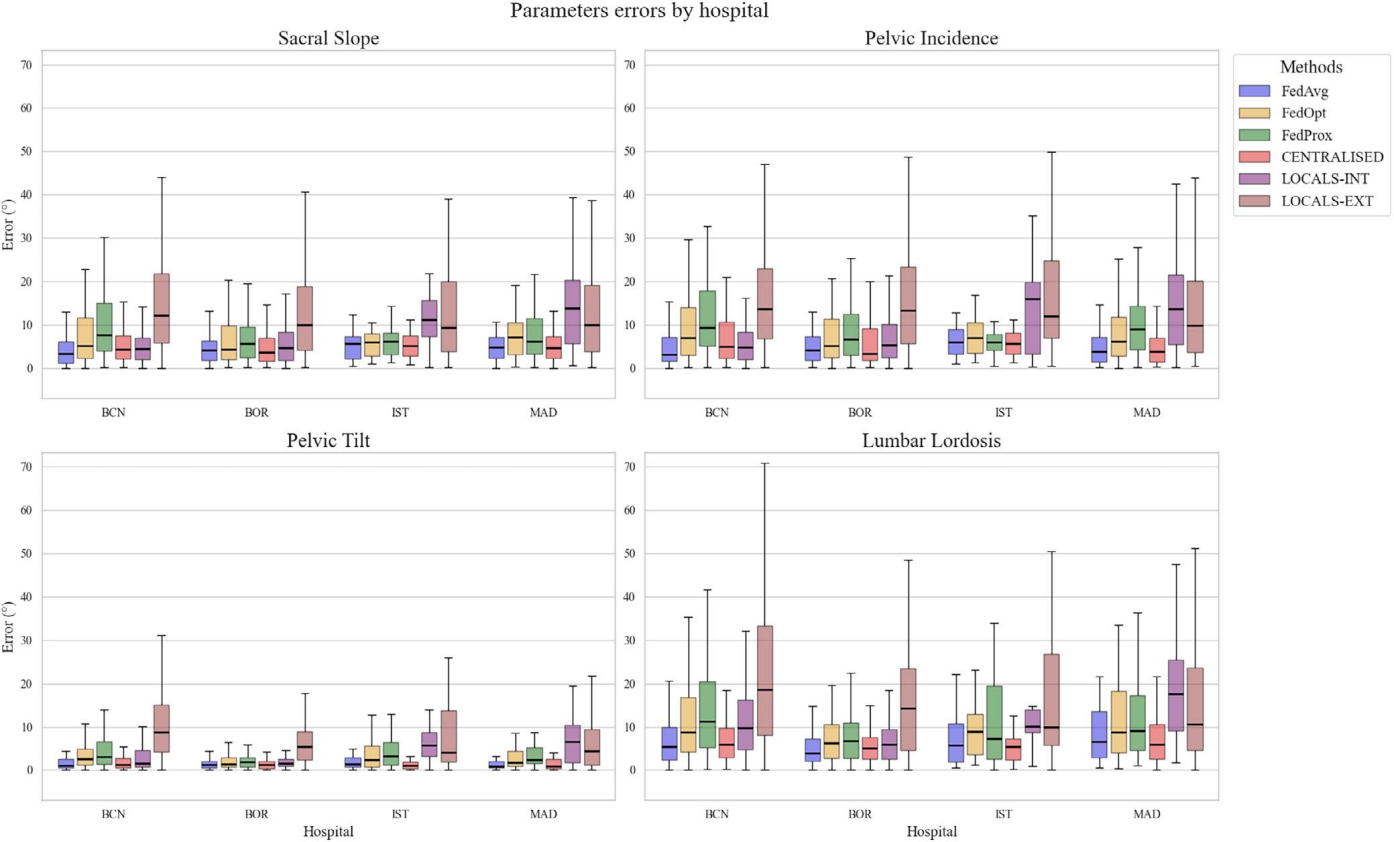
Parameter errors by hospital. Boxplots illustrate the estimation errors (in degrees) for each of the four parameters (sacral slope, pelvic incidence, pelvic tilt, and lumbar lordosis) stratified by hospital location (BCN, BOR, IST, and MAD). The four training methods are compared side by side to highlight how each approach performs within individual hospital datasets.

The hospital‐specific analysis aligns with these findings that FedAvg is not inferior to centralized training. Moreover, in hospitals with larger datasets, such as BCN and BOR, local models evaluated on internal data performed very similarly to centralized and FedAvg models and significantly better than local models externally validated (Figure 6). For example, for the SS parameter in BCN, the centralized model yielded a mean error of 6.86° (SD = 10.10°), FedAvg achieved 5.05° (SD = 5.98°), and the local model tested internally produced 5.87° (SD = 7.48°). Similarly, in BOR, the corresponding errors were 6.55° (SD = 13.98°) for the centralized model, 4.87° (SD = 4.61°) for FedAvg, and 6.51° (SD = 7.04°) for the internally tested local model. However, in hospitals with fewer images (e.g., IST and MAD), local models exhibited substantially higher error rates even when tested on internal data. For SS, IST's internal error was 11.14° (SD = 6.52°) and MAD's was 16.49° (SD = 16.33°), compared to centralized errors of 5.15° (SD = 2.88°) and 7.04° (SD = 11.60°), and FedAvg errors of 5.42° (SD = 3.78°) and 6.93° (SD = 11.20°) in IST and MAD, respectively. In general, FedAvg maintained performance very similar to that of the centralized model, outperforming local models in hospitals with a limited number of images. The local models tested externally always performed worse. Although FedOpt and FedProx underperformed compared to FedAvg, they still appear beneficial for smaller hospitals (e.g., MAD and IST) by outperforming local models on internal tests. An even more detailed overview of each hospital's performance when externally validated can be seen in Figure S1.

#### 3.2.4 Percentage of Correct Predictions

**FIGURE 7:**
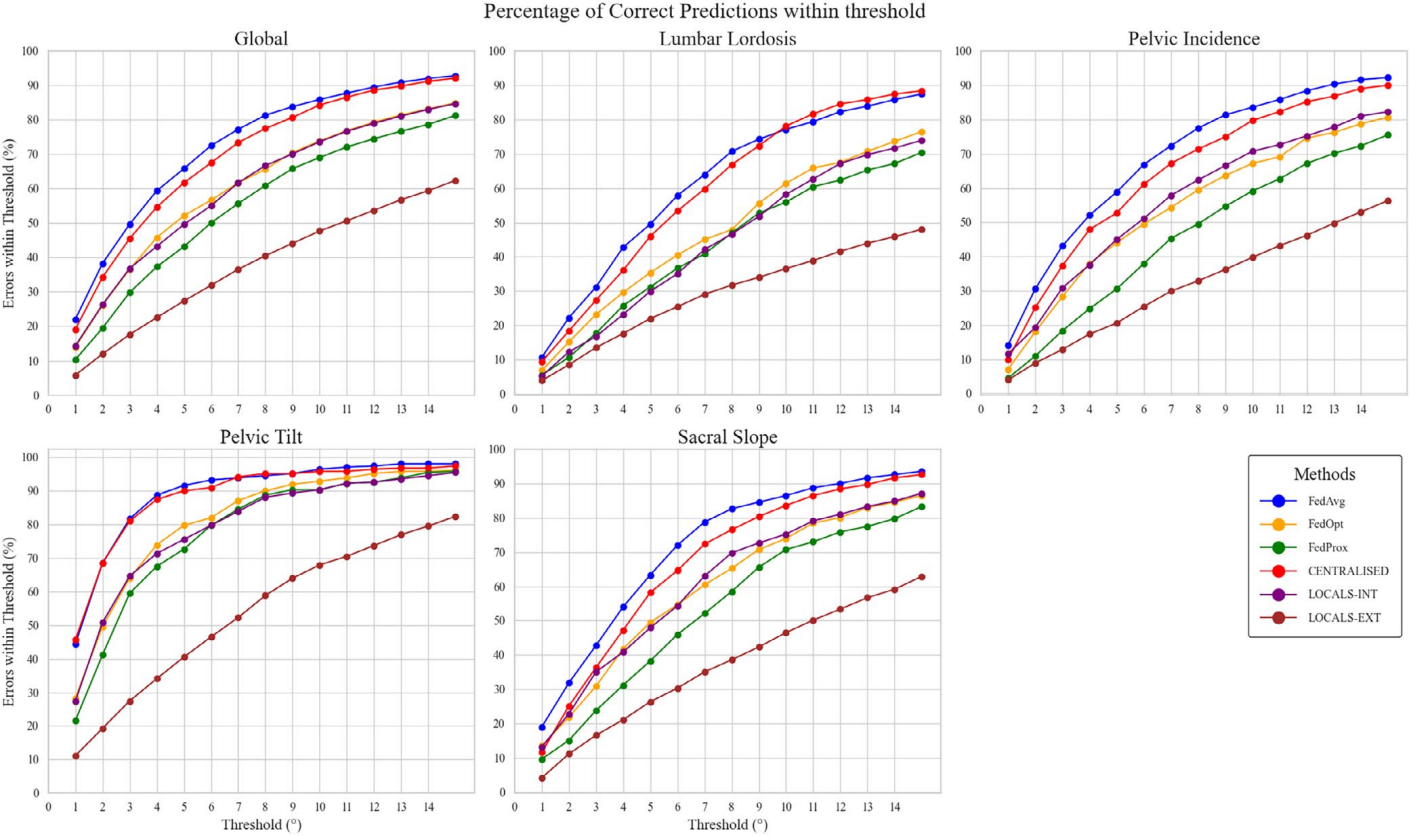
Percentage of correct predictions. Percentage of predictions within varying error thresholds (1°–15°) for anatomical angles (lumbar lordosis, pelvic incidence, pelvic tilt, and sacral slope) and globally averaged performance across angles, comparing federated (FedAvg, FedOpt, FedProx), centralized (centralized), and locally trained (LOCALS‐INT, LOCALS‐EXT) models.

Prediction accuracy was further evaluated by calculating the percentage of angle estimates falling within error thresholds from 1° to 15°. FedAvg consistently outperformed all other methods, with ~70% of predictions within 5° of the ground truth, followed closely by the centralized model (~67%). FedOpt and FedProx achieved moderate performance (~60% and ~55%, respectively), while local models lagged significantly, capturing only ~48% (internal) and ~27% (external) of predictions within the same threshold. These trends held across all individual anatomical parameters, underscoring the robustness and generalizability of federated and centralized approaches over site‐specific models (Figure 7).

#### 3.2.5 Comparison of FedAvg, FedOpt, and FedProx

Focusing solely on the federated approaches, FedAvg consistently outperforms both FedOpt and FedProx across all parameters. For example, in predicting SS, FedAvg gives a mean absolute error of 5.27° (SD = 6.46°), compared to 7.87° (SD = 10.27°) for FedOpt and 9.11° (SD = 9.30°) for FedProx. This pattern is even more pronounced for PI, where the errors are 5.84° for FedAvg, versus 8.87° and 11.12° for FedOpt and FedProx, respectively. Similar trends are observed for PT (2.19° for FedAvg vs. 3.64° and 4.28°) and LL (7.08° for FedAvg vs. 10.94° and 12.52°). Overall, while all FL approaches provide benefits, particularly in data‐limited settings, FedAvg stands out as the most robust, offering superior prediction accuracy and generalizability. This is likely due to its straightforward and balanced aggregation of information across hospitals, which enhances model stability and improves its ability to manage variability across patient populations.

## 4 Discussion

This study aimed to simulate an FL approach for estimating lumbosacral alignment parameters from lumbar x‐ray images and to compare its performance against a centralized model and independent local models. The centralized approach represents an ideal scenario, as pooling data from all hospitals should theoretically yield the most robust model due to the diversity and completeness of the dataset. However, real‐world constraints such as data privacy concerns, regulatory hurdles, and the complexities of data‐sharing agreements often make centralized training impractical [24, 38]. In contrast, FL offers a promising alternative by enabling collaborative training without requiring raw data exchange, thereby maintaining patient privacy while still taking advantage of multi‐institutional data.

FL is gaining traction across a range of clinical domains, offering a privacy‐preserving alternative to traditional data centralization. In oncology, one landmark study demonstrated that aggregating model updates from 71 institutions worldwide enabled robust and generalizable glioblastoma boundary detection, overcoming barriers to data sharing and enhancing tumor delineation accuracy [22]. Another approach, ProxyFL, introduced a decentralized FL framework that combines proxy models with differential privacy to enable efficient, peer‐to‐peer collaboration. This method allows each institution to retain control over its model architecture while maintaining strong performance and reducing communication overhead [39]. Always related to oncology, a study proposed FL‐PedBrain, an FL‐based AI platform for pediatric brain tumors, showing that federated training across 19 international sites can achieve performance comparable to centralized approaches in both tumor classification and segmentation while significantly boosting performance at sites with smaller datasets [23]. Most recently, a dedicated review on FL in spine surgery [26] highlighted its potential to address critical challenges related to data privacy and heterogeneity in spine‐focused predictive modeling.

This study represents one of the first attempts to simulate a FL approach in the spine domain, specifically examining its feasibility for calculating clinically relevant lumbopelvic parameters. A recent study [40] proposed a FL‐based vertebral segmentation framework (FLVBSF) employing Dual Attention Gates (DAGs) to collaboratively train accurate segmentation models across multiple institutions, effectively addressing data scarcity and privacy concerns in spinal imaging tasks. Our findings demonstrate that FedAvg achieves comparable or slightly superior performance to the centralized model, with mean absolute errors for key parameters consistently within a similar range. These findings are in line with those of reported previous works showing that FL does not typically underperform relative to centralized training, highlighting its suitability for building high‐performing models while preserving patient privacy. The marginally better performance of FedAvg may reflect a bias in centralized training, where institutions with larger datasets exert disproportionate influence during optimization. In contrast, independently trained local models—particularly when externally validated—consistently underperform, underscoring their limited generalizability. FL mitigates this limitation by aggregating knowledge across centers, thereby enhancing robustness and reducing degradation during external validation [24, 41]. Notably, FL models outperformed even locally trained models evaluated on internal data, especially in hospitals with smaller datasets (e.g., MAD and IST). The higher internal errors at these two centers were likely due to two factors. First, smaller per‐site training sets increase variance and risk of overfitting. Second, a within‐center distribution shift is possible: protocols and equipment change over time, positioning and field of view can vary across operators, and the patient‐level split could concentrate different cases in the internal test subset. With few patients, these differences were not fully covered during training, leading to poorer internal poorer performance. Our findings illustrate how FL can mitigate these problems and enable smaller institutions to benefit from collaborative training, significantly improving predictive performance (Figure 6).

We evaluated three FL strategies: FedAvg, FedOpt, and FedProx. Despite its simplicity, FedAvg consistently outperformed FedOpt and FedProx in our experiments. While this does not imply FedAvg is inherently superior, its straightforward configuration—primarily the number of local epochs and FL rounds—makes it highly attractive for practical deployment and broader experimentation. In contrast, FedOpt and FedProx require tuning of additional hyperparameters (e.g., learning rates, momentum decay, and regularization terms for FedProx), which demand deeper domain expertise in FL. In our experience, these configurations introduced sensitivity, with performance occasionally suffering due to suboptimal convergence or local minima. FedAvg, by contrast, demonstrated stable performance across settings, making it a pragmatic and robust baseline for researchers exploring FL applications in healthcare. This superior performance might be further explained also by the greater robustness of FedAvg under few participating hospitals. Noisy, heterogeneous client updates were mitigated by simple averaging, whereas server‐side optimizers in FedOpt could amplify update noise and might be sensitive to server learning rate and momentum choices. The proximal term in FedProx could stabilize training, but under limited FL rounds and fixed local epochs, it may slow useful local progress if its influence is too strong or offer little benefit if the influence is too weak. Therefore, in our practical setting, FedAvg showed more stable behavior across centers.

Despite these encouraging results, the study has several limitations. First, the experimental design involved limited hyperparameter tuning and assessed only a single network architecture, leaving the potential benefits of alternative architectures or optimized configurations unexplored. Moreover, the scope of FL strategies examined was narrow, excluding several state‐of‐the‐art methods that could offer performance or efficiency gains. For example, FedScaffold [42] mitigates client drift by aligning local updates with server directions, potentially improving convergence and model stability, while FedGKT [43] leverages knowledge transfer to reduce communication overhead, which may enhance scalability in resource‐constrained settings. Additionally, our simulation assumed synchronous participation from all client hospitals—an idealized condition that may not reflect real‐world FL deployments where intermittent connectivity or limited computational resources can hinder update cycles. Finally, neither the centralized nor FL‐trained models underwent a proper external validation on truly independent datasets, limiting conclusions about their broader generalizability. External validation is a mandatory step in clinical settings and will be addressed in future works. Future research should also involve a more in‐depth analysis of FL approaches and a detailed exploration of hyperparameters for the FL approaches based on server‐side optimization as well as the use of more advanced neural network architectures developed in recent years.

Therefore, to fully establish FL as a robust and clinically viable approach, future research must extend validation to real‐world, multi‐institutional settings where client availability, computational heterogeneity, and network instability present practical challenges. Addressing these factors will be essential to ensure that FL systems not only sustain high predictive performance but also function reliably across diverse clinical environments. In addition, validating the models on data from a truly independent external institution will further strengthen evidence for their generalizability. These steps are critical to advancing FL as a practical, privacy‐preserving framework for collaborative medical imaging analysis in spine research.

## Conflicts of Interest

Catherine R. Jutzeler serves as a scientific consultant to Abbvie and Mitsubishi Takeda. Yann Philippe Charles is a consultant to Stryker and Clariance. Ahmet Alanay serves as a consultant to DePuy Synthes, Medtronic, Highridge, and Globus. Caglar Yilgor is a consultant for Medtronic. Ferran Pellisé is a consultant for Medtronic, Johnson & Johnson Medtech, Globus‐Nuvasive, SpineArt, and Orthofix. However, the consultant roles did not influence the design, conduct, or reporting of this study. The other authors declare no conflicts of interest. The European Spine Study Group receives funding support from DePuy Synthes and Medtronic. However, the two companies had no influence on the design, conduct, or reporting of this study.

## Supporting information


**Figure S1:** Heatmaps showing prediction errors (in degrees) for each anatomical parameter (sacral slope, pelvic incidence, pelvic tilt, and lumbar lordosis) across individual hospitals (BCN, BOR, IST, MAD). Results are compared between locally trained models (columns BCN, BOR, IST, MAD), centralized training (centralized), and federated learning strategies (FedAvg, FedOpt, FedProx). Darker colors indicate higher prediction errors. For each heatmap, a vertical white line separates the local models' performance from the centralized and FL approaches.
**Data S1:** Supporting information.

## Data Availability

The datasets generated and/or analysed during the current study are not publicly available due to privacy and ethical restrictions. The code to run the analysis as well as create the figures is available on our GitLab repository (https://gitlab.ethz.ch/BMDSlab/publications/low‐back/federatedlearning).
