# Supplementary material for: Simulating Federated Learning to Enable Multi‐Hospital Collaboration for Lumbopelvic Alignment Estimation
Source: JOR Spine. 2025 Oct 16;8(4):e70120. doi: 10.1002/jsp2.70120 (PMC12529873; doi:10.1002/jsp2.70120)
Supplement: Supplementary file 1 — Figure S1: Heatmaps showing prediction errors (in degrees) for each anatomical parameter (sacral slope, pelvic incidence, pelvic tilt, and lumbar lordosis) across individual hospitals (BCN, BOR, IST, MAD). Results are compared between locally trained models (columns BCN, BOR, IST, MAD), centralized training (centralized), and federated learning strategies (FedAvg, FedOpt, FedProx). Darker colors indicate higher prediction errors. For each heatmap, a vertical white line separates the local models' performance from the centralized and FL approaches. Data S1: Supporting information. [file JSP2-8-e70120-s001.zip › jsp270120-sup-0002-Data S1.docx]

The heatmaps provide a detailed view of how the different models perform on each hospital separately (Figure S1). In these heatmaps, the rows indicate the test hospital and the columns represent the source of the training model (centralised, federated, or local). For example, the position corresponding to the row ‘BOR’ and the column ‘MAD’, represents the performance (in terms of mean absolute error for a specific parameter) of the local model trained on MAD and externally tested on BOR. Going into more detail, it is clear that centralised and FedAvg approaches outperformed the models trained on single hospital data, particularly if they were tested on external hospitals. Moreover, all federated learning approaches (FedAvg, FedOpt, and FedProx) demonstrated a clear advantage for hospitals with limited data; for instance, when considering the prediction of PI, local models from IST and MAD gave mean absolute errors of 14.2° and 14.7° on their own test sets, while the federated approaches reduced these errors to 6.9° (IST) and 6.0° (MAD) for FedAvg, 10.1° (IST) and 9.2° (MAD) for FedOpt, and 6.9° (IST) and 10.8° (MAD) for FedProx.

**Figure S1:** Heatmaps showing prediction errors (in degrees) for each anatomical parameter (Sacral Slope, Pelvic Incidence, Pelvic Tilt, and Lumbar Lordosis) across individual hospitals (BCN, BOR, IST, MAD). Results are compared between locally trained models (columns BCN, BOR, IST, MAD), centralised training (CENTRALISED), and federated learning strategies (FedAvg, FedOpt, FedProx). Darker colors indicate higher prediction errors. For each heatmap, a vertical white line separates the local models' performance from the centralised and FL approaches.
