# Supplementary figures and images for: Simulating Federated Learning to Enable Multi‐Hospital Collaboration for Lumbopelvic Alignment Estimation
Source: JOR Spine. 2025 Oct 16;8(4):e70120. doi: 10.1002/jsp2.70120 (PMC12529873; doi:10.1002/jsp2.70120)

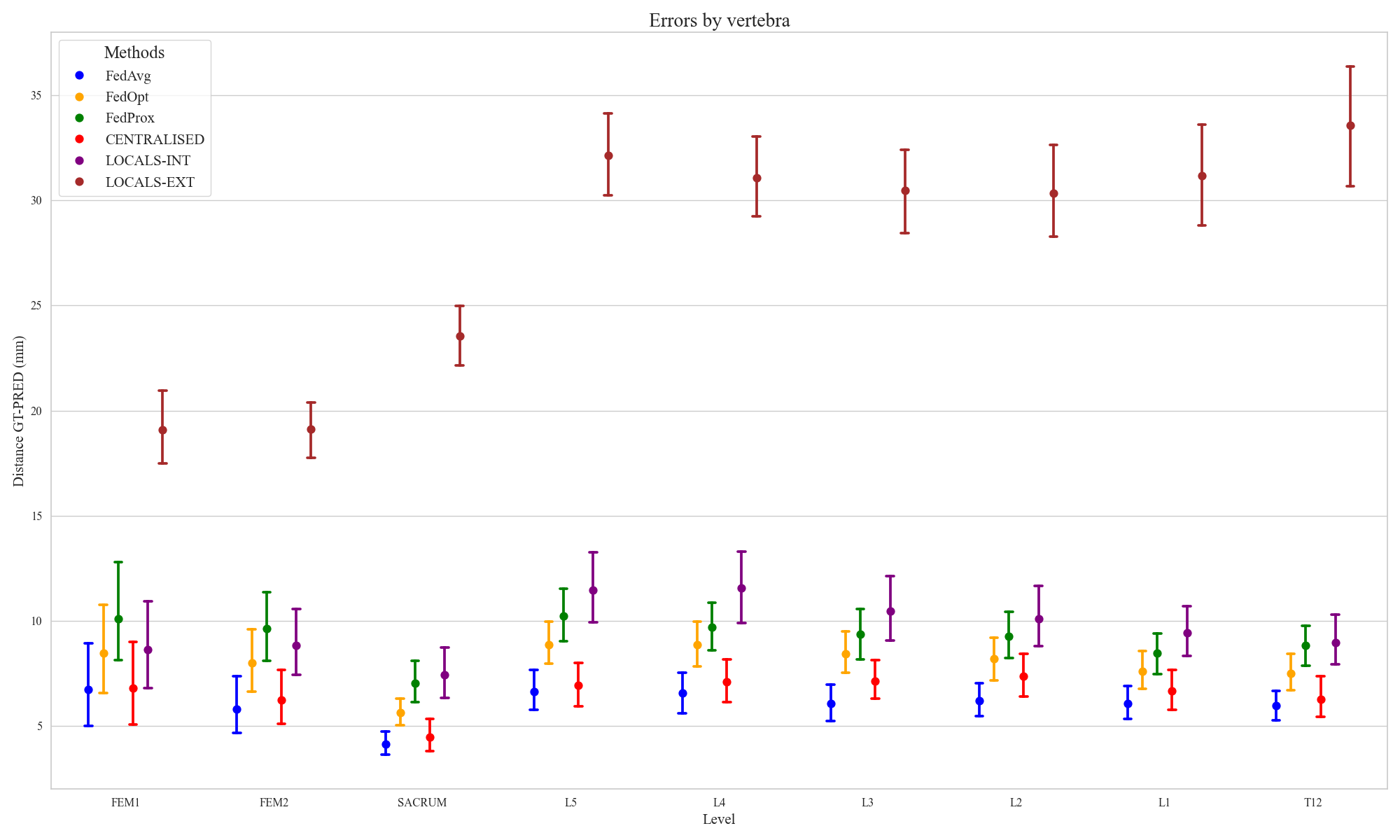

Supplement: Supplementary file 1 — Figure S1: Heatmaps showing prediction errors (in degrees) for each anatomical parameter (sacral slope, pelvic incidence, pelvic tilt, and lumbar lordosis) across individual hospitals (BCN, BOR, IST, MAD). Results are compared between locally trained models (columns BCN, BOR, IST, MAD), centralized training (centralized), and federated learning strategies (FedAvg, FedOpt, FedProx). Darker colors indicate higher prediction errors. For each heatmap, a vertical white line separates the local models' performance from the centralized and FL approaches. Data S1: Supporting information. [file JSP2-8-e70120-s001.zip › jsp270120-sup-0001-FigureS1]
